# An analysis of 30 years of surface ozone concentrations in Austria: temporal evolution, changes in precursor emissions and chemical regimes, temperature dependence, and lessons for the future†

**DOI:** 10.1039/d2ea00004k

**Published:** 2022-04-19

**Authors:** Monika Mayer, Stefan F. Schreier, Wolfgang Spangl, Christoph Staehle, Heidelinde Trimmel, Harald E. Rieder

**Affiliations:** Institute of Meteorology and Climatology, Department of Water, Atmosphere, and Environment (WAU), University of Natural Resources and Life Sciences (BOKU) Vienna Austria monika.mayer@boku.ac.at; Environment Agency Austria Vienna Austria

## Abstract

Despite substantial reductions in anthropogenic emissions of nitrogen oxides (NO_*x*_) and non-methane volatile organic compounds (NMVOCs) in Austria over the 30 year time period 1990–2019, summertime surface ozone (O_3_) concentrations still exceed frequently and over wide areas the ozone maximum 8 hour mean target value for the protection of human health. We present a detailed analysis of *in situ* observations of O_3_ and NO_*x*_ to (1) disentangle the main processes propelling O_3_ formation such as precursor emissions and meteorology and (2) quantify the impact of NO_*x*_ reductions and (3) estimate the effect of climate warming. The temperature sensitivity of surface O_3_ production is assessed separately for NO_*x*_ and VOC limited regimes. The temperature sensitivity of ozone increases with temperature in spring and summer. On average, the evaluated absolute values of the sensitivities are a factor of 2.5 larger in summer than in spring. The analysis of ambient O_3_ burdens during hot summers indicates that rising temperatures in a warming climate might largely offset the benefit of future emission reductions. MAX-DOAS formaldehyde (HCHO) measurements in Vienna from 2017 to 2019 are used as a proxy for VOC emissions. The seasonal and the temperature dependence of the observed HCHO mixing ratios indicate that biogenic VOCs (BVOCs) are the dominant source of hydrocarbons in the urban setting during the ozone season. The result agrees well with VOC emission estimates that show BVOCs to be the dominant VOC source in Austria since the early 2000s. Accordingly, anthropogenic NO_*x*_ emission reductions remain, outside of urban cores, the most effective instrument for policymakers to lower surface ozone concentrations in the short term.

## Abstract 2

We analyse the evolution of surface ozone in Austria for 1990–2019 and investigate effects of changes in precursor emissions and climate warming. Our results show that ozone burdens remain a health problem despite reductions in precursor emissions.

## Graphical abstract

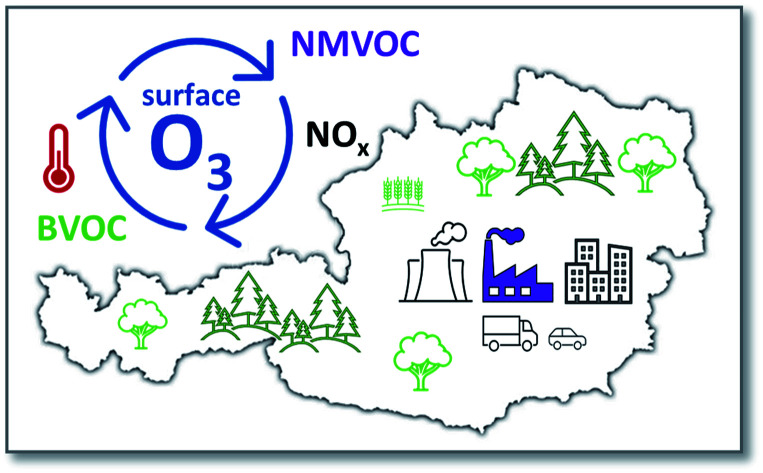

**Environmental significance**

Elevated surface ozone concentrations continue to be of concern for public health in many countries, despite substantial reductions of anthropogenic precursor emissions during recent years. Progressive climate warming accompanied by increased biogenic volatile organic compound emissions propels ozone production. On a regional basis the contribution of changes in individual factors affecting surface ozone concentrations such as anthropogenic and natural emissions, solar radiation or temperature is not well constrained. In our work, we quantify the effect of NO_*x*_ emission reductions, and the temperature sensitivity of surface ozone based on 30 years of observational data for NO_*x*_ and VOC limited chemical regimes in Austria. Overall, we find that the climate penalty outweighs the gains achieved by emission reductions. Applying our analysis scheme would allow other research teams to investigate the climate penalty on ozone formation in other countries/world regions. Furthermore, our results provide a first-order estimate regarding the magnitude of changes in the surface ozone burden that can be expected in light of climate warming and emission changes over the next few decades, thereby also providing a context for results obtained with chemistry–climate and chemistry–transport models.

## 1 Introduction

Surface ozone is harmful at elevated levels in surface air to human health^1–4^ and vegetation, *e.g.* ref. 5–7, and also impacts surfaces of built structures.^8^ Furthermore, O_3_ is an important tropospheric greenhouse gas belonging to the group of short-lived climate forcers.^9–12^ The global average radiative forcing due to increases in tropospheric O_3_ since pre-industrial times is estimated to be +0.35 ± 0.15 W m^−2^.^13^ While the average atmospheric lifetime of free tropospheric O_3_ is in the order of weeks,^14^ it reduces to a few hours for surface O_3_ in areas with elevated precursor emissions, *e.g.* ref. 15, leading to a highly variable spatial and temporal ozone burden.

In surface air, O_3_ is produced through a series of chemical reactions involving sunlight and O_3_ precursors such as nitrogen oxides (NO_*x*_: NO and NO_2_), non-methane volatile organic compounds (NMVOCs), carbon monoxide (CO) and methane (CH_4_).^15^ These precursors are emitted from anthropogenic (*e.g.*, traffic or industry) and natural (*e.g.*, soil, trees, lightning) sources, *e.g.* ref. 4. Ozone production rates depend strongly on the chemical environment (*i.e.*, NO_*x*_ or VOC limitation) and ambient meteorological conditions (*i.e.*, solar radiation, temperature, and humidity).^15,16^ The O_3_ loss occurs through deposition at the surface and uptake by plants, heterogeneous reactions involving aerosols, or O_3_ titration. The latter occurs close to combustion sources, *e.g.* in urban environments, during the night when excess amounts of NO react with O_3_ to form NO_2_, *e.g.* ref. 17. The highest O_3_ concentrations are typically found in or downwind of major municipalities, *e.g.* ref. 16, and not in the urban core (due to the aforementioned titration effects). Mountain sites also generally exhibit elevated O_3_ concentrations because of enhanced short-wave radiation, intrusions of stratospheric O_3_, *e.g.* ref. 18 and 19, and an increased chemical lifetime of O_3_ in the free atmosphere.

As surface O_3_ is harmful to human health, national governments have implemented the European ozone air quality legislation in recent decades.^20^ The compliance with air quality standards is evaluated with monitoring networks applying state-of-the-art precision measurement techniques. The European Union (EU) defines an O_3_ target value and a long-term objective for protecting human health in the directive 2008/50/EC.^21^ The ozone target value for the protection of human health is defined as the maximum daily eight-hour average value (MDA8), not to exceed 120 μg m^−3^ on more than 25 days per calendar year averaged over three years. Although the EU MDA8 target value lies well above the World Health Organization's (WHO's) air quality guideline (*i.e.*, MDA8 not to exceed 100 μg m^−3^), it is frequently not attained in Austria despite substantial efforts to curb precursor emissions, and the subsequent reductions achieved.^22^ During the 3 year evaluation period, 2017–2019, the EU target value was exceeded at 57% of the Austrian monitoring sites on more than 25 days per year.^23^ Furthermore, in 2019, the 120 μg m^−3^ threshold value was violated at least once at every station in Austria.

To understand changes in surface O_3_, it is essential to disentangle the factors influencing O_3_ concentrations. Important factors are anthropogenic and biogenic precursor emissions, the meteorological conditions that affect precursor dispersion, and photochemistry. Further, the temperature dependence of surface ozone production is relevant in the context of global warming. Today it is well understood that ozone chemistry depends nonlinearly on the above-mentioned drivers.^15^

In this paper, we focus on the effect of reduced precursor emissions (particularly NO_*x*_ emissions) and increasing temperatures (in spring and summer) on Austrian surface O_3_ concentrations during 1990–2019. We exclude data for the year 2020, given the widespread impact of COVID-19 related restrictions on anthropogenic emissions and thus ambient air quality in Austria. Our findings provide a comprehensive characterisation of the evolution of the past O_3_ burden in Austria. They also enable us to estimate potential future developments in surface O_3_ burdens in the light of projections of further declining NO_*x*_ emissions^22^ and concurrent rising temperatures due to climate change.^24,25^

## 2 Data and methods

### 2.1. Air quality data and metrics

Nationwide air pollution monitoring, including O_3_ and NO_*x*_, started around 1990 in Austria. The current monitoring network is operated by the Environment Agency Austria (Umweltbundesamt) and the governments of the nine Austrian provinces.

For surface O_3_ the monitoring network comprised in 2019 a total of 99 background sites in rural (50), suburban (40) and urban (9) regions, as well as additional monitors at traffic (6) and industrial sites (4) (note that monitoring sites are classified according to the guidelines of the European Environment Agency, EEA).^26^ The highest short-term O_3_ values (1 hour mean values) are observed in the densely populated region around the capital Vienna; the highest long-term levels (8 hour-mean values, annual mean values) are recorded in medium and high altitude alpine regions.^23^

For the present analysis, we consider only background sites located at altitudes below 1500 m a.s.l. and stations that were continuously operated throughout 1990–2019. This selection yields a total of 54 background sites (6 urban, 20 suburban, and 28 rural), for which we calculate the MDA8 O_3_.

To address the issue of meteorological variability and its impact on surface ozone formation, we aggregate data over 10 year periods,^27^ resulting in three time periods 1990–1999, 2000–2009, and 2010–2019. This enables us to study changes (in the order of several μg m^−3^) that are smaller than the inter-annual fluctuations caused by the variability of ambient meteorology. We analyse for each monitor category (1) the number of days on which the 120 μg m^−3^ EU threshold for MDA8 O_3_ is exceeded on an annual basis and for the warm seasons (JJA and MAM) of the year, which show peak ozone abundance, and (2) the spring and summertime probabilistic 1-, 3-, and 5-year return levels (RLs) of MDA8 O_3_. To this end we fit a peak-over-threshold model,^28^ with the threshold *u* = 120 μg m^−3^, to seasonal MDA8 O_3_ data for the decadal time slices defined above. These *n*-year RLs represent the MDA8 O_3_ values, exceeded in probabilistic terms at least once in *n*-years over the decadal time slice considered.^29^ Furthermore, we investigate the impact of temperature (a climate penalty) on MDA8 O_3_ by contrasting results for full 10 year time periods and the same time periods excluding data of hot years.

Along with ozone, we analyse co-located measurements of NO and NO_2_ available from the Austrian air quality monitoring network. Continuous observations are available at 39 background sites during 1990–2019 (5 urban, 18 suburban, and 16 rural sites). For these data, we focus hereinafter on the daily average (DA) NO_*x*_ mixing ratio (expressed in ppb).

**Fig. 1:**
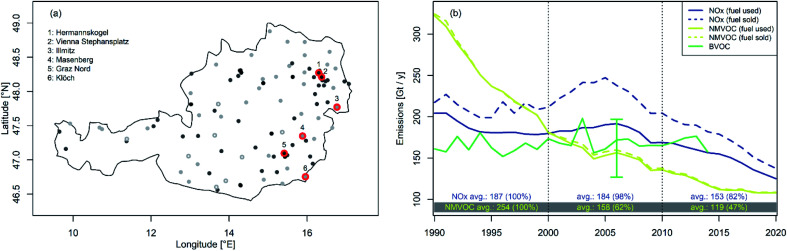
(a) Overview of the location of background O_3_ monitoring sites (light grey refers to rural, dark grey to suburban and black to urban sites). Numbered sites marked with red circles are selected as representative examples for site categories. (b) Estimates of Austrian NO_*x*_, NMVOC and biogenic VOC emissions in 1990–2019. Blue numbers at the bottom of the panel indicate the ratio of NO_*x*_ emissions, and yellow numbers indicate that of NMVOC emissions, in a particular 10 year time period relative to the base period 1990–1999.

A table of all O_3_ and NO_*x*_ stations used in this study is provided in the ESI in Table S1.†Fig. 1a shows the location of the selected ozone monitoring sites; here, circles with a white dot indicate sites with co-located NO_*x*_ measurements. For illustrative purposes, we select another six representative stations (highlighted with red circles in Fig. 1a and marked with an asterisk in Table S1:† one urban, two suburban and three rural sites, located in or in the vicinity of Austria's largest municipalities Vienna and Graz). We use these sites to illustrate the chemical processes driving O_3_ formation and decay in more detail.

By analysing the co-evolution of O_3_ (MDA8) and NO_*x*_ (DA) under clear sky conditions we determine whether O_3_ chemistry is NO_*x*_ or VOC limited. If the observed MDA8 O_3_ and DA NO_*x*_ data are directly proportional, we deduce a NO_*x*_ limited regime. If O_3_ reveals no correlation with NO_*x*_, we find O_3_ chemistry to be VOC limited.^30^ The distinction between the chemical regimes can only be achieved if the total amount of global radiation is large enough not to limit ozone production. For both chemical regimes, peak ozone is known to be temperature sensitive, but to different extents.^31^ Therefore, we include mean temperature (*T*_mean_) as a covariate to obtain the temperature sensitivity of MDA8 O_3_ separately for spring and summer (see Section 4.3).

Formaldehyde (HCHO) is a short-lived oxidation product of VOCs (including methane – CH_4_) and can therefore be used as a proxy for VOC emissions, *e.g.* ref. 32 and 33. The HCHO background concentration is determined by the oxidation of CH_4_,^34,35^ a globally well-mixed trace gas, and other long-lived VOCs.^33^ Observed variations of HCHO concentrations originate either from VOC oxidation processes (secondary source) or primary HCHO emissions (mainly combustion processes). BVOCs are highly reactive, resulting in a short atmospheric lifetime in the order of hours.^36,37^ As a result, ambient HCHO concentrations are substantially influenced on short time and small spatial scales by BVOC emissions. Today it is well understood that BVOCs are an important HCHO source, even in urban areas.^38^ The partitioning between secondary and primary sources in urban areas is about 95% to 5%, *e.g.* ref. 39 and 40.

Since June 2017, HCHO has been measured continuously in Vienna with a series of MAX-DOAS instruments that are part of the VINDOBONA network (https://www.doas-vindobona.at/).^41^ Here we use data retrieved from the instrument, stationed on the roof of the University of Natural Resources and Life Sciences, Vienna (BOKU). We analyse MAX-DOAS retrievals in the UV wavelength range (338–370 nm) for a light path that stretches horizontally across Vienna's city center (median path length is 9 km) at an altitude of approximately 150 m above ground for solar zenith angles smaller than 75°. We then calculate from the available daytime retrievals the DA HCHO mixing ratio.

HCHO MAX-DOAS observations are further combined with DA NO_*x*_, MDA8 O_3_ and *T*_mean_ data for Vienna's city center (Stephansplatz) to investigate O_3_ chemistry at the urban background site. This enables us to derive O_3_ isopleths directly from observations, using HCHO as a proxy for VOCs. Additionally, we examine the relationship between *T*_mean_ and HCHO mixing ratios. HCHO observations in Vienna allow us to focus on three objectives, (1) to confirm our approach to determining the O_3_ chemical regime, (2) to derive an ozone isopleth plot purely from observations, and (3) to estimate the impact of BVOCs in the largest metropolitan region in Austria.

### 2.2. Evolution of precursor emissions

In tandem with emissions in most European regions, Austrian anthropogenic NO_*x*_ and VOC emissions dropped significantly between 1990 and 2019.^4,42^Fig. 1b provides an overview of the Austrian national annual total anthropogenic emissions of NO_*x*_ and NMVOCs over the last few decades.^22^ The gridded data we analyse here are available at the EMEP Centre on Emission Inventories and Projections (https://www.ceip.at/the-emep-grid/gridded-emissions). BVOC emissions are derived from various models such as Bauwens *et al.* (2018)^43^ for isoprene (https://emissions.aeronomie.be; BIRA IASB, 2018) and Simpson and Winiwarter (1998)^44^ for other BVOCs. Two data sets for NO_*x*_ and NMVOCs are given: one provides emission estimates based on fuel sold (dashed line) and one based on fuel used (solid line). The latter is used as a reference in our analysis as it represents the better proxy for actual emissions in Austria. We note that the difference between the two scenarios is minimal for NMVOCs since traffic is not a dominant source. Austrian NO_*x*_ emissions decreased between 1990 and 2019 by about 35%, while NMVOC emissions decreased by about 66%. If we analyse average NO_*x*_ emissions over the 10 year periods, we find in 2000–2009 a decline of only 2% and in 2010–2019 a decline of 18% compared to the reference time-period 1990–1999 (for NMVOCs: −33% in 2000–2009 and −53% in 2010–2019, respectively).

NMVOC emissions reached in 2002 approximately the same level as estimated BVOC emissions (see Fig. 1b). The error bar given in Fig. 1b for the year 2006 is derived from the work of Skjoth *et al.* (2008)^45^ and Oderbolz *et al.* (2013).^46^ The isoprene share of BVOC emissions was determined by a modelling effort driven by reanalysis data and constrained by HCHO satellite observations.^32,43^ Monoterpenes and oxygenated volatile organic compound emissions are assumed to be constant over time.^44^ Since 2014 BVOC emissions have been thought to be twice as large as national anthropogenic NMVOC emissions.^22^ Concerning surface O_3_, VOC emissions during spring and summer are most relevant. It was estimated that about 80% of the annual emissions occur during summer.^33,47–49^ Future Austrian anthropogenic NMVOC emissions are projected to reach levels slightly lower than today,^22,50^ while BVOC emissions are likely to increase due to their temperature dependence, *e.g.* ref. 48, 51 and 52. As Austrian NO_*x*_ emissions are expected to decrease substantially in the coming decades,^53,54^ it is crucial to understand the response of surface ozone concentrations to these reductions while anthropogenic NMVOC emissions remain essentially unchanged. Hence, we focus explicitly on the time-period 2010–2019, for which we assess ozone's temperature sensitivity at rural, suburban, and urban sites.

To investigate the correlation between annual emission estimates and observed DA NO_*x*_ mixing ratios during the prime ozone season (March to August) we analyse the ECDFs (empirical cumulative distribution functions) of measured DA NO_*x*_. The median of observed DA NO_*x*_ mixing ratios decreased across all monitor categories by 30–37% during the spring and summer season in 2010–2019 compared to 1990–1999. This decline in the 10 year average observed DA NO_*x*_ mixing ratio is larger than the corresponding emission reduction (−18%). In the ESI in Fig. S1,† we show the ECDFs of observed DA NO_*x*_ mixing ratios for spring (MAM) and summer (JJA) for 1990–1999, 2000–2009 and 2010–2019. For completeness, Table S2 in the ESI† lists the observed changes for the 50th and 90th quantiles for each site category for MAM and JJA, respectively.

### 2.3. Meteorological data

As meteorological covariates, we consider air temperature (2 m above ground) and total global radiation (*i.e.*, the daily sum of global radiation), available from gridded data sets (SPARTACUS (1 km × 1 km grid)^55,56^ and APOLIS (100 × 100 m grid)^57^) provided by the Austrian meteorological service Zentralanstalt für Meteorologie und Geodynamik (ZAMG). Given the importance of photochemistry for O_3_ formation, we specify radiation levels by selecting clear sky days when investigating the impact of NO_*x*_ and temperature on O_3_ concentrations. To quantify radiation, we calculate the daily total global radiation for each monitoring site. The annual cycle of the daily sums of the total global radiation is plotted, and the envelope of the 30 years of observational data is fitted with a polynomial of the 5th order. This envelope-function approximates our maximum feasible total global radiation for each day of the year. “Clear sky” conditions are assumed if the observed total daily global radiation reaches 90% of the polynomial fit on a specific day. The selection of clear sky days indicates that mean and maximum temperatures are correlated. We choose the daily mean temperature (*T*_mean_) at the site level from the gridded data set as variable because we anticipate a significant variance in the ozone productivity solely due to the difference in prevailing temperatures in spring and summer.

## 3 Results

### 3.1. Evolution of MDA8 O_3_ between 1990 and 2019

**Fig. 2:**
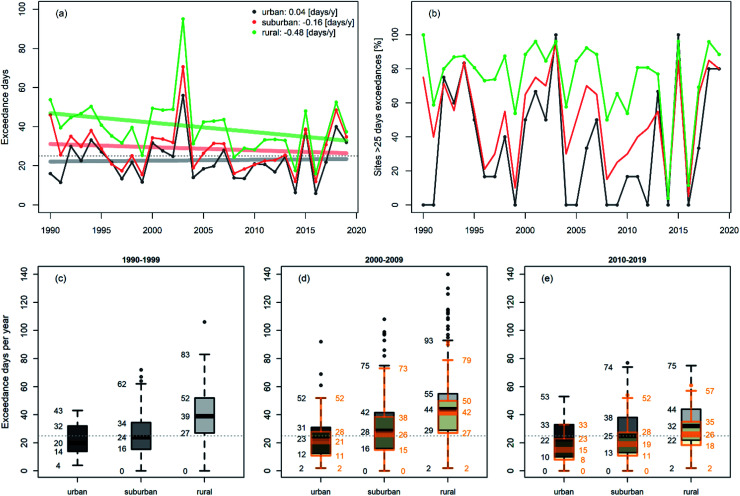
(a) Annual average number of exceedance days of the MDA8 O_3_ threshold value of 120 μg m^−3^ for each category of monitoring sites in Austria. (b) The percentage of sites for each category that exceeded. (c–e) Boxplots of the annual number of exceedance days of the MDA8 O_3_ threshold value for urban, suburban, and rural background sites for 1990–1999, 2000–2009 and 2010–2019. Boxplots in orange colour in (d) and (e) exclude measurements for 2003 and 2015, 2017, 2018, and 2019, respectively. The colour-coded solid lines in panel (a) provide linear trend estimates per site category. The dotted line in panels (a) and (c)–(e) indicates the target threshold of 25 days.

While hourly peak O_3_ values have substantially decreased over the last three decades,^23^ exceedances of the MDA8 O_3_ threshold of 120 μg m^−3^ are still regularly observed nationwide, particularly during the summer season. Fig. 2a illustrates the time evolution of the annual average number of exceedance days of the MDA8 O_3_ target value for the protection of human health for urban, suburban, and rural monitoring sites. The fewest exceedances are observed at urban sites, the most at rural stations. Overall exceedances at all site categories show a negative trend between 1990 and 2019. Despite some difference in the precise time periods considered, our results show a strong agreement with those provided by Chang *et al.* (2017)^58^ for European sites contained in the tropospheric ozone assessment report (TOAR) database. In Fig. 2b, we plot the percentage of sites exceeding the MDA8 O_3_ in each category. The apparent large year to year variability can be attributed to different meteorological conditions. The high number of exceedance days in 2003, 2015, 2017, 2018 and 2019 is related to the five hottest summers (JJA) in Austria since the beginning of national records in 1767 (deviation in summer temperature relative to the average for 1981–2010 is in 2003: +2.8 °C, 2019: +2.7 °C, 2015: +2.4 °C, 2017: +2.1 °C, and 2018: +2.0 °C (ref. 59)). As high temperatures propel ozone production, their occurrence is directly reflected by an increased number of days above the MDA8 O_3_ threshold value. To study the influence of hot seasons such as the aforementioned hot summers, we exclude these years in an additional assessment, *i.e.* for 2000–2009 we omit 2003, and for 2010–2019 we omit 2015, 2017, 2018, and 2019, respectively.

In Fig. 2c–e, we show the spread in the annual average number of exceedances per site location category (urban, suburban, and rural background) for all three selected time periods. The evaluations without data for the hot years are plotted in orange, respectively. Most outliers (at all site location categories) in the MDA8 O_3_ exceedances during 2000–2009 and 2010–2019 occurred during hot summers (Fig. 2d and e).

Overall, we find an increase in exceedance frequencies at all monitoring stations during 2000–2009 compared to the 1990–1999 period. At rural monitors, we observe a decrease in 2010–2019 (on average minus 7 exceedance days) relative to the base period, whereas at suburban and urban sites, the average number of exceedance days converged to the limit value of 25 days. If we omit years with hot summers from our analysis (orange boxplots in Fig. 2d and e), the number of exceedance days in the period 2000–2009 is slightly higher compared to 1990–1999 but substantially lower in the period 2010–2019 (median: minus 5 days at suburban and urban, minus 13 days at rural sites). The actual magnitude of the change in surface O_3_ is defined by the underlying ozone production mechanisms. These are mainly VOC limited in central urban areas, and NO_*x*_ limited in rural areas during summer. The NO_*x*_ to VOC ratio is predominantly altered during heat waves by increased BVOC emissions caused by the heat stressed plants (*e.g.* observed isoprene mixing ratios in the UK were found to be a factor of 4 higher during heat spells^60^). Heatwaves are commonly accompanied by stagnant, clear sky conditions, leading to elevated radiation levels under which photochemical reactions are accelerated. Furthermore, under such conditions, O_3_ dry deposition typically diminishes because plants close their stomata and reduce therefore the O_3_ uptake.^61^

At rural monitoring sites, *i.e.*, in NO_*x*_ limited areas, we expect NO_*x*_ emission reductions to translate directly into a reduction in the number of exceedance days. The observed q50 of DA NO_*x*_ mixing ratios at rural sites was reduced by 36% between 1990–1999 and 2010–2019 (see Table S2†) during the ozone season. The number of days with MDA8 O_3_ exceedances decreased for the final analysis period by 18% (minus 7 days) at rural sites reflecting the adverse impact of a warming climate. If we exclude years with hot summers, the median of exceedance days reduces in 2010–2019 by an additional 6 days, yielding a total of minus 13 days (−33%). This reduction is very close to the 36% decrease in DA NO_*x*_ mixing ratios that we derive from measurements at rural sites.

Precursor concentrations at suburban sites represent the transition zone between NO_*x*_ and VOC limited regimes, depending on pollutant transport (enhanced NO_*x*_ concentrations downwind of urban cores) and VOC levels. The similarity of the evolution of exceedance days at suburban and urban monitors indicates that peak MDA8 O_3_ concentrations occur primarily in the VOC limited regime.

To study the time evolution of the MDA8 O_3_ and not only exceedances of 120 μg m^−3^, we investigate spring (March, April, May – MAM) and summer (June, July, August – JJA) separately. O_3_ chemistry may differ seasonally at a particular site due to the impact of BVOCs with their seasonal cycle. Changes in the seasonal MDA8 O_3_ burden mimic the temporal evolution of exceedance days discussed above. On average, surface ozone concentrations are elevated in 2000–2009 for both seasons compared to 1990–1999 throughout all categories of background sites. Increasing O_3_ abundances in 2000–2009 have been followed by an overall decline of MDA8 O_3_ concentrations in 2010–2019 in MAM as well as JJA. The median MDA8 O_3_ levels at rural sites in the last decadal time slice match concentrations observed in the base period (in spring and summer). For suburban and urban monitors, we find slight increases in the median values in the order of 2–5 μg m^−3^ (4 μg m^−3^ in JJA for suburban and urban, 5 μg m^−3^ in MAM for urban, and 2 μg m^−3^ in MAM for suburban sites) between the periods 1990–1999 and 2010–2019. This rise in the MDA8 O_3_ median values is primarily driven by increases in the lower tail of the MDA8 O_3_ distribution. In contrast, changes in the upper tail are less pronounced (see Fig. S2 in the ESI† for the evolution of the MDA8 O_3_ concentrations for MAM and JJA).

At all site categories, the range of concentrations decreased in the middle and last 10 year time period with respect to the first. This can be attributed to the reduction of precursor emissions resulting in narrower O_3_ distribution functions (see Fig. S2,† where the length of whiskers in the boxplots becomes shorter with time). The most pronounced peak MDA8 O_3_ reductions are found at rural sites where ozone production is NO_*x*_ limited (maximum MDA8 O_3_ ∼minus 15 μg m^−3^ in both seasons in the last period compared to the first period). The analysis without hot years allows us to attribute changes linked to higher temperatures. We find an increase of the median MDA8 O_3_ of about 2 μg m^−3^ in 2000–2009 and about 6 μg m^−3^ in 2010–2019 because of the enhanced ozone productivity at elevated summertime temperatures. In spring the temperature effect is much smaller, 0–1 μg m^−3^ in 2000–2009 and 1–3 μg m^−3^ in 2010–2019 despite spring temperatures in 2003, 2015, 2017, and 2018 being well above the climatological mean of 1981–2010 (2003: +1.5 °C, 2015: +1.5 °C, 2017: +2.4 °C, 2018: +2.7 °C, 2019: +0.4 °C (ref. 59)). Elevated temperatures affect peak ozone during spring to a much smaller extent than during the summer season because the whole temperature range is too low for efficient ozone production. A detailed temperature sensitivity analysis of MDA8 O_3_ is presented in Section 4.3.

To assess changes in peak MDA8 O_3_ in more detail, we apply methods from extreme value theory (EVT) in the next section.

### 3.2. Extreme value analysis – return levels of MDA8 O_3_

Since summer months are most prone to high surface O_3_ levels, we focus in the extreme value assessment on MDA8 O_3_ during JJA. Applying a peak-over-threshold (POT) model with a threshold of 120 μg m^−3^, we derive the probabilistic MDA8 O_3_ return levels at intervals of 1, 3, and 5 years.

**Fig. 3:**
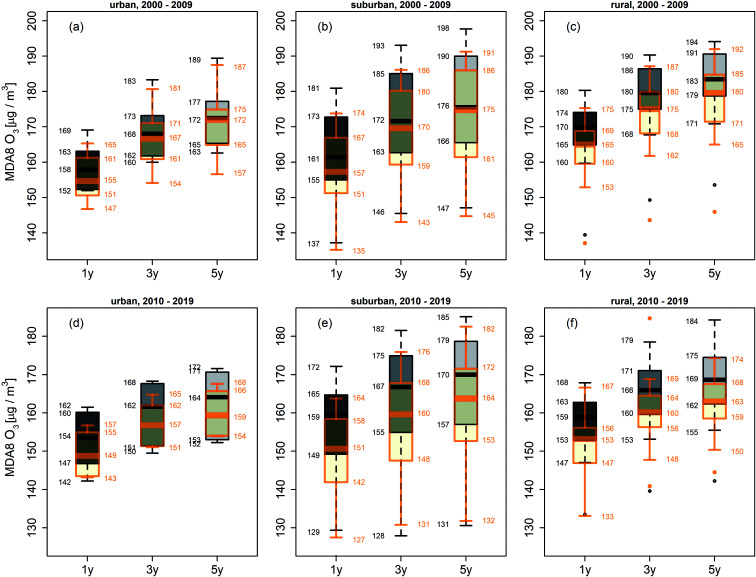
Boxplots of 1-, 3-, and 5-year return levels for JJA MDA8 O_3_ in 2000–2009 for (a) urban, (b) suburban and (c) rural sites. (d)–(f) as (a)–(c) but for 2010–2019. Boxplots in orange colour refer to the analysis without the hot years: 2003 (a–c) and 2015, 2017, 2018, and 2019 (d–f).

The RLs corresponding to the MDA8 O_3_ value exceeded at least once in 1, 3 or 5 years, respectively, thus illustrating the changes in peak ozone abundances. In Fig. 3, boxplots of the estimated summertime MDA8 O_3_ RLs for urban, suburban, and rural sites are shown for 2000–2009 and 2010–2019. The median RLs are generally highest in rural areas, while suburban sites show the largest spread in site-level RLs.

To estimate the impact of heatwaves on MDA8 O_3_ RLs, we analyse and contrast MDA8 O_3_ RLs for 2000–2009 and 2010–2019, including/excluding the years with hot summers (2003, 2015, 2017, 2018, and 2019). In Fig. 3, the orange boxplots indicate the analysis without the extreme years with generally smaller MDA8 O_3_ RLs. The 1 year RLs are lowered on average by ∼4 μg m^−3^ in 2000–2009 and by ∼6 μg m^−3^ in 2010–2019 when excluding hot years. This illustrates the temperature penalty on O_3_ during hot summers. The median 1 year RLs of the decadal time slices decreased by ∼4 μg m^−3^ at urban, by ∼2 μg m^−3^ at suburban and by ∼11 μg m^−3^ at rural sites in 2010–2019 compared to 2000–2009. For higher-order return levels, decreases particularly at rural and suburban sites are even more pronounced, yielding up to ∼6 μg m^−3^ for 3 year RLs and ∼14 μg m^−3^ for 5 year RLs.

## 4 Relationship between MDA8 O_3_, DA NO_*x*_, total global radiation, and daily mean surface temperature

As we focus on peak O_3_ concentrations, we study ozone chemistry only under clear sky conditions (see the definition in Section 2.3). Observations of NO_*x*_ mixing ratios, total global radiation, and mean temperature allow us to investigate ozone chemistry at the site level and its temperature dependence in greater detail. The subsequent analysis is performed on a seasonal basis for MAM and JJA for the period from 2010 to 2019, as this time period is most informative for potential future changes in surface ozone following emission controls. During this period, annual anthropogenic NMVOC emissions decreased slightly and approached a value comparable to (near-)future estimates.^22^ In a first approximation, we can assume anthropogenic NMVOC emission to be constant on an annual basis. Hence the analysis of this period allows us to study the impact of NO_*x*_ reductions on ozone concentrations which will be relevant for future scenarios. First, we select 6 representative sites (see Fig. 1a, red circled stations): one urban, two suburban, and three rural sites in close proximity in two Austrian regions, to demonstrate the approach. Subsequently, we use the results of all monitors to generate a contour plot of MDA8 O_3_ temperature sensitivity with respect to observed NO_*x*_ mixing ratios and *T*_mean_.

### 4.1. MDA8 O_3_*vs.* DA NO_*x*_

**Fig. 4:**
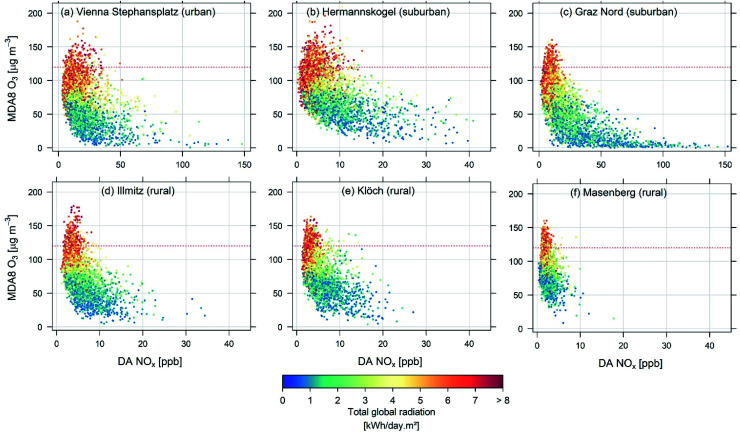
Scatterplots of MDA8 O_3_*versus* DA NO_*x*_ for (a) the urban station Vienna Stephansplatz, (b and c) suburban sites Hermannskogel and Graz Nord, and (d–f) rural sites Illmitz, Klöch, and Masenberg for 2010–2019. The red dotted line in all panels indicates the MDA8 O_3_ threshold value of 120 μg m^−3^. Colour coding is according to daily total global radiation abundances.

Scatterplots of (all year) MDA8 O_3_ and DA NO_*x*_ data for 2010–2019 are shown in Fig. 4 for selected sites, colour coded according to radiation abundances. Note the wide range in DA NO_*x*_ mixing ratios within each site category, specifically for suburban and rural sites. Generally varying mixing layer heights determine NO_*x*_ mixing ratios at each site throughout the year. The red dotted line in all panels indicates the EU threshold value of 120 μg m^−3^ for MDA8 O_3_.

Across sites, we find enhanced ozone production if total global radiation exceeds 4 kW per day per m^2^ (3.46 MJ m^−2^, yellow to red data points). Conversely, for low radiation values (blue to green data points), the observed MDA8 O_3_ is determined by the ambient O_3_ background concentration, or O_3_ is even found to be depleted if the monitor is close to NO_*x*_ emission sources (titration). O_3_ titration is seen at the urban site Vienna (Fig. 4a) and the suburban site Graz (Fig. 4d) for days with total global radiation below 2 kW per day per m^2^ (1.73 MJ m^−2^).

### 4.2. Clear sky MDA8 O_3_ chemistry

**Fig. 5:**
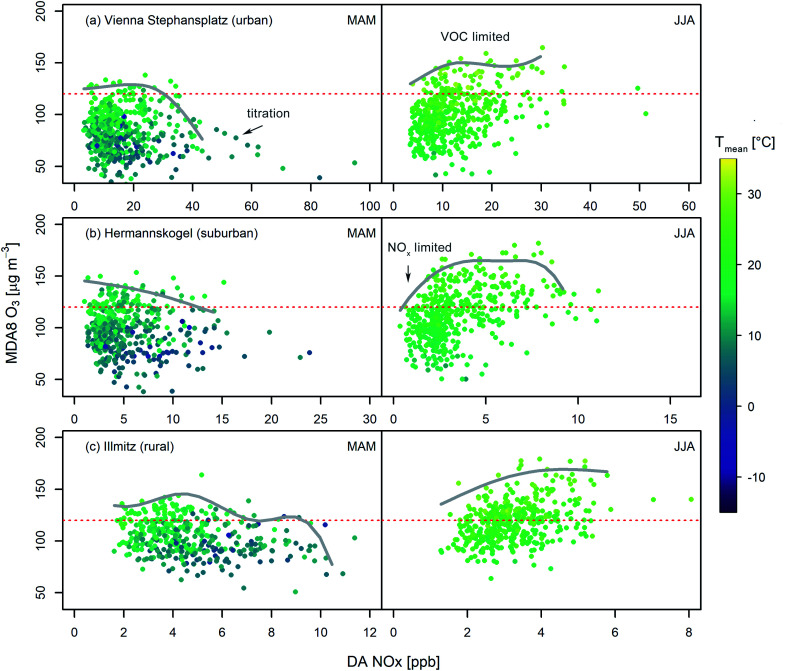
MDA8 O_3_*versus* daily average (DA) NO_*x*_ during summer (JJA) and spring (MAM) under clear sky conditions for (a) the urban station Vienna Stephansplatz, (b) the suburban site Hermannskogel, and (c) the rural site Illmitz. The red dotted line in all panels indicates the threshold of 120 μg m^−3^. MDA8 O_3_ is coloured according to *T*_mean_. The grey lines are polynomial fits of the 4th order to the binned data envelopes.

We filter measurements with respect to clear sky conditions to assess if ozone chemistry is NO_*x*_ or VOC limited. In the case of NO_*x*_ limited chemistry, we expect ozone concentrations to follow NO_*x*_ proportionally. Conversely, for VOC limited conditions, we anticipate no relationship between O_3_ and NO_*x*_.^30^ For both regimes, ambient temperature is an important covariate. Fig. 5 shows the seasonal (MAM and JJA) scatterplot of MDA8 O_3_*versus* DA NO_*x*_, colour-coded by *T*_mean_, for an urban (Vienna Stephansplatz), a suburban (Hermannskogel), and a rural (Illmitz) site for 2010–2019. The corresponding illustrations for the other 3 exemplary stations – Graz Nord, Klöch, and Masenberg – are given in ESI Fig. S3.†

To indicate the chemical regimes, splines are calculated as polynomial fits of the 4th order to the envelopes of the scatterplots of MDA8 O_3_*versus* DA NO_*x*_. For the envelope fitting, we bin the observations in 20 equally sized bins using data between the 10% and 90% quantiles (to avoid introducing arbitrary effects by outliers) and fit to bin maxima. The splines obtained are plotted in Fig. 5 (and in the ESI in Fig. S3†) as grey lines. For the urban station in Vienna Stephansplatz (Fig. 5a), three domains based on NO_*x*_ levels can be identified during spring: low-range NO_*x*_ (DA NO_*x*_ < 8 ppb) which is NO_*x*_ limited, medium-range NO_*x*_ (8 ppb < DA NO_*x*_ < 40 ppb) which is VOC limited, and high-range NO_*x*_ (DA NO_*x*_ > 40 ppb) where ozone concentrations decline with increasing NO_*x*_ (ozone titration because of relatively low absolute radiation in early spring). During summertime, ozone chemistry is predominantly VOC limited. In this regime, the highest ozone concentrations are observed. Only for DA NO_*x*_ below 8 ppb NO_*x*_ limitation occurs (∼20% of the observations). Generally, rising temperatures facilitate enhanced O_3_ production, *i.e.*, for a given DA NO_*x*_ value the MDA8 O_3_ yield increases with rising temperature.

For suburban sites, we expect a mix of VOC and NO_*x*_ limited conditions from theory. Fig. 5b provides seasonal scatterplots of MDA8 O_3_*versus* DA NO_*x*_ for the monitoring site Hermannskogel. Ozone production during MAM appears mainly VOC limited (4 ppb < DA NO_*x*_ < 18 ppb). However, at very low NO_*x*_ concentrations (DA NO_*x*_ < 4 ppb) the chemical regime shifts towards NO_*x*_-limitation. In summer, NO_*x*_ concentrations are found to be significantly lower than in MAM. As a result, NO_*x*_ limited ozone chemistry becomes more important. About 65% of the observations reside in the NO_*x*_ limited regime in JJA. In Fig. 5c, seasonal scatterplots for the rural station Illmitz are shown. Here a NO_*x*_ limitation of ozone chemistry is observed during summertime, while NO_*x*_ limitation emerges in spring only for very low NO_*x*_ mixing ratios (<4 ppb). For DA NO_*x*_ greater than 5 ppb, ozone chemistry is VOC limited, indicating the limited availability of BVOCs at that time of the year.

**Fig. 6:**
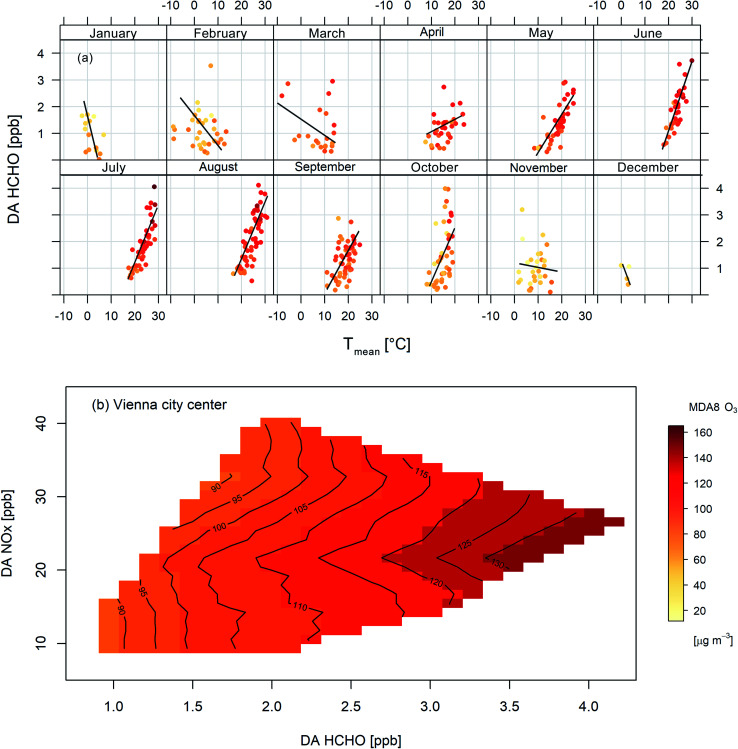
(a) Temperature (*T*_mean_) dependence of observed HCHO mixing ratios in Vienna (city centre) colour-coded according to MDA8 O_3_ for the period from 06/2017 to 12/2019. (b) MDA8 O_3_ isopleth plot for MAM and JJA derived from path averaged DA HCHO mixing ratios (approx. 9 km across Vienna's city centre) and *in situ* O_3_ and NO_*x*_ measurements.

For the metropolitan area of Vienna, MAX-DOAS HCHO observations (starting 06/2017) are available. This data allows us to analyse the correlation of HCHO mixing ratios and *T*_mean_. If BVOC emissions are the dominant source, observed HCHO mixing ratios are expected to rise with temperature during the growing season. In Fig. 6a, we plot DA HCHO mixing ratios *versus T*_mean_ for clear sky days (time-period 06/2017–12/2019). Data are fit with a linear model. From April to October, observed MDA8 O_3_ concentrations increase with temperature. From November to March, ozone and HCHO are negatively correlated. Colder days and lower mixing layer heights result in higher HCHO mixing ratios. This illustrates that BVOCs control hydrocarbon chemistry in Vienna's city centre during the growing season, which overlaps with the ozone season. The HCHO measurements allow us also to investigate ozone chemistry directly by assessing ozone isopleth plots. Spring and summertime measurements of HCHO (MAX-DOAS), NO_*x*_, and O_3_ (*in situ*, urban site Stephansplatz) are combined to obtain the O_3_ isopleth plot displayed in Fig. 6b. This illustration clearly reveals that peak MDA8 O_3_ concentrations are in the VOC limited regime. Most of the data employed for the isopleth plot stem from hot springs and summers (with average temperature lying more than 2 °C above the climatological mean for 1981–2010, except spring 2019, which was only +0.4 °C warmer). Hence the isopleths may provide some guidance regarding estimates for future ozone chemistry in Vienna in a warming climate.

### 4.3. MDA8 O_3_ temperature sensitivity

The temperature sensitivity of the observed surface ozone concentrations is predominantly determined by the temperature sensitivity of chemical kinetics and the temperature dependence of BVOC emissions. Increased BVOC concentrations due to higher temperatures will lead to enhanced ozone concentrations in VOC limited areas, *e.g.* ref. 62, such as the Vienna metropolitan region. Based on our results for Vienna, we assume BVOCs to be the dominant source in the other urban environments across Austria.

The temperature dependence of the ozone kinetics is determined by the absolute NO_*x*_ concentration.^31^ It is weak for the low NO_*x*_ range (NO_*x*_ limited) and pronounced for the VOC limited range. For both regimes, a non-linear relation between ozone production and temperature is observed. To determine ozone's temperature sensitivity, we plot MDA8 O_3_*versus T*_mean_ for each site for clear sky days and fit the data with a linear model (LM). The linear fit works well because we distinguish between seasons, MAM and JJA, respectively. Therefore, the whole temperature range is partitioned into segments, and within each segment, *T*_mean_ and MDA8 O_3_ show a linear correlation. For convenient reference, we provide scatterplots with the linear fits for the 6 sample stations in ESI Fig. S6† for MAM and JJA. LM slopes are generally larger in JJA than in MAM, the broadest range of observed temperature sensitivities (2.5 to 5 μg per m^3^ per °C *T*_mean_) is found at suburban sites in JJA, and the highest values occur at urban sites in JJA (6 μg per m^3^ per °C *T*_mean_).

**Fig. 7:**
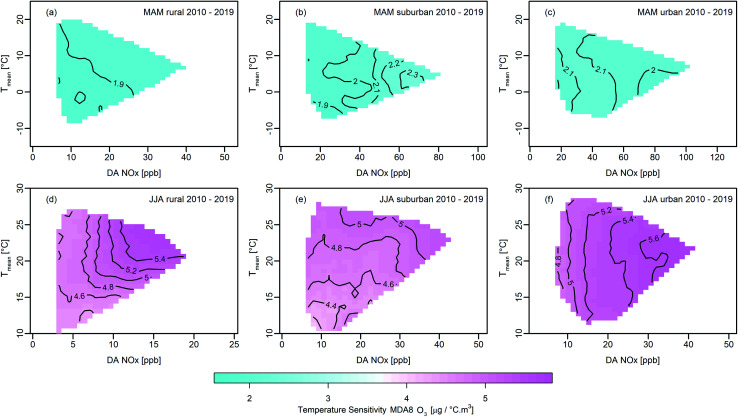
MDA8 O_3_ temperature sensitivities for rural, suburban, and urban monitors for various DA NO_*x*_ mixing ratios and mean temperatures. The ozone temperature sensitivities for 2010–2019 under clear sky conditions are shown for spring (MAM) in the top panels (a–c) and for summer (JJA) in the bottom panels (d–f). The contours of the temperature sensitivity of ozone are given in μg °C^−1^ m^−3^ with respect to changes of *T*_mean_. Plots for 1990–1999 and 2000–2009 can be found in ESI Fig. S8 and S9.†

In the next step, the temperature sensitivity of MDA8 O_3_ defined by the LM slopes is related to the median of the corresponding DA NO_*x*_ mixing ratios. The contour plots in Fig. 7 summarise the slopes of the linear fits – changes of the MDA8 O_3_ in μg °C^−1^ m^−3^ – for both seasons for rural, suburban, and urban sites for 2010–2019. In spring, the temperature sensitivity is much smaller than in summer (∼2 μg °C^−1^ m^−3^). During summer, absolute values at all site categories are found in the range of 4.4–5.6 μg °C^−1^ m^−3^ but reveal a very different correlation with respect to DA NO_*x*_ mixing ratios.

At rural sites under low NO_*x*_ conditions (DA NO_*x*_ < 5 ppb), the MDA8 O_3_ sensitivity is constant for all observed *T*_mean_ values, whereas for DA NO_*x*_ > 5 ppb, the temperature sensitivity increases with *T*_mean_. In the low NO_*x*_ range, as defined by Vogel *et al.* (1999),^31^ the dominant temperature-sensitive reaction is the release of NO_2_ from peroxyacetyl nitrate (PAN). In our study, rural monitoring sites can be predominately ascribed to the low NO_*x*_ range. Hence, the observed temperature sensitivity at rural sites can be understood by considering the decomposition of PAN, and the VOC to NO_*x*_ ratio changes due to increasing BVOC emissions with rising temperatures.

The altered NO_*x*_/VOC ratio with the additional BVOCs leads to a higher ozone production rate and therefore enforces the effect of PAN decomposition. For suburban monitors, the temperature sensitivity generally increases with temperature, reflecting the temperature dependence of VOC dominated chemistry^31,63^ and the temperature dependence of BVOC emissions. The observed DA NO_*x*_ mixing ratios for suburban monitors (>5 ppb) also indicate mainly VOC limited ozone chemistry, as illustrated in Fig. 5b for Hermannskogel, and Fig. S3a† for Graz Nord. The strongest increase in the temperature sensitivity (25%) is observed at rural sites with DA NO_*x*_ mixing ratios ranging from 5 ppb to 20 ppb. For *T*_mean_ ∼10 °C, the sensitivity is found to be 4.4 μg °C^−1^ m^−3^ and rises for *T*_mean_ ∼20 °C to 5.4 μg °C^−1^ m^−3^ in summer.

A one °C increase in *T*_mean_ results on average in a 5 μg m^−3^ increment in MDA8 O_3_. This is a substantial increase, particularly considering the decrease of MDA8 O_3_ median concentrations achieved due to precursor emission reductions since 1990, which are of the same order of magnitude (2–5 μg m^−3^ when hot years are excluded from the analysis, see Fig. S2d–f†). Within the last 30 years, Austrian NO_*x*_ concentrations declined by ∼30–40%. Simultaneously, NMVOC emissions are estimated to have decreased by ∼60%. As illustrated in Fig. 2e, the series of hot summers in the period from 2010 to 2019 offset almost completely the MDA8 O_3_ reductions obtained by decreasing precursor emissions. Our results indicate that the (MDA8) O_3_ temperature sensitivity will pose a continuing challenge for air pollution regulators in suburban and urban environments, where the ozone production regime is VOC limited and (temperature-dependent) BVOCs are nationwide the dominant VOC source.

In the ESI in Fig. S8 and S9,† the temperature sensitivity of ozone is provided for the time periods 1990–1999 and 2000–2009. While we do not find a pronounced change in ozone-temperature sensitivity patterns for the individual site categories, we find a decrease in the overall magnitude of the ozone-temperature sensitivity in spring and an increase in summer. This is particularly pronounced in spring, where absolute values decreased from ranging between 1.9 and 2.8 μg °C^−1^ m^−3^ in 1990–1999 to 0.8 and 1.4 μg °C^−1^ m^−3^ in 2010–2019. Ozone production is predominantly VOC limited in spring. Thus, the reduction of anthropogenic NMVOC emissions propels less efficient ozone production regimes, which reflects also in the decrease of the overall temperature sensitivity of chemical ozone production.

As in spring, temperature sensitivity patterns do not change during summer over time except for urban sites in 2000–2009, which can be related to altered NO_*x*_ to VOC ratios during that time (*i.e.*, more significant reduction in NMVOCs compared to NO_*x*_). In absolute terms, the temperature sensitivity increased over time during summer by about 10% at urban sites.

## 5 Discussion and conclusions

In this study, we present an analysis of 30 years of ozone observations available from rural, suburban, and urban background sites in Austria. Our analysis focuses on days with MDA8 O_3_ exceeding the national and the European Union's target threshold for the protection of human health. To reduce the noise stemming from meteorological variability in the analysis, we assess changes over three 10 year time periods covering the years 1990–1999, 2000–2009 and 2010–2019. MDA8 O_3_ concentrations larger than 120 μg m^−3^ are still frequently observed at all site categories (rural, suburban, and urban) during spring and summer despite substantial precursor emission declines: NO_*x*_ emissions were cut by 18% (observed concentrations declined by ∼30–40%) and anthropogenic NMVOC emissions by 53% in 2010–2019 compared to 1990–1999.

We investigated several factors affecting MDA8 O_3_ to understand the changes occurring in the past and assess possible future developments. We studied the modified VOC to NO_*x*_ ratios emerging from diverse reductions of anthropogenic precursors over the 30 year study time-period. Altered precursor ratios explain the different impacts of emission abatements at various site categories. Our results show that during summer (JJA), ozone production is predominantly NO_*x*_ limited at rural sites and VOC limited at urban sites. Furthermore, for suburban environments, we identify over time a shift in the ozone chemical regime towards NO_*x*_ limitation. Focusing on the MDA8 O_3_ distribution, the most significant reduction of the upper tail (75% quantile, q75) MDA8 O_3_ is found comparing 2010–2019 to 1990–1999 at (NO_*x*_ limited) rural sites (q75 minus 8 μg m^−3^*vs.* suburban (urban) sites: q75 minus 4 (4) μg m^−3^). Similarly, the largest reduction of exceedance days is found at rural sites (minus 7 exceedance days), while slight increases are found at suburban (and urban) sites (plus 1 (2) exceedance days). The narrowing of the MDA8 O_3_ probability distribution function is caused by lower anthropogenic NO_*x*_ and VOC emissions. As a result, MDA8 O_3_ maxima decreased while minima increased simultaneously. The extent of the site-level changes depends on the prevailing ozone chemistry. In the VOC limited regime, the MDA8 O_3_ median increased between the initial and the final time period by 4 μg m^−3^ (initially at 94 (96) μg m^−3^ for suburban (urban) sites). At NO_*x*_ limited rural sites, the median remained unchanged at 104 μg m^−3^, and overall rural sites continued to show the highest ozone concentrations across all site categories.

Concerning the temperature sensitivity of ozone production, our study shows that the temperature sensitivity of MDA8 O_3_ is one of the main drivers of elevated ozone levels. Increasing temperatures have been boosting ozone production to such an extent that in recent hot summers (2015, 2018, and 2019) the number of annual exceedance days (MDA8 O_3_ > 120 μg m^−3^) was well above the target of 25 days at all site categories. While we do not find a pronounced change in ozone-temperature sensitivity patterns for the individual site categories, we see a decrease in the overall magnitude of the ozone-temperature sensitivity in spring and an increase in summer. The analysis shows further that the temperature increase annihilated the benefits from emission reductions. The latter is clearly seen when we repeat our analysis and exclude hot summers. For MDA8 O_3_, we find a decrease in the median, peak values (q75), and exceedance days at all site categories (rural: median −5 μg m^−3^, q75 −16 μg m^−3^, exceedance days −13; suburban/urban: median −2 μg m^−3^, q75 −14 μg m^−3^, exceedance days −5) in 2010–2019 compared to 1990–1999.

Given the strong temperature sensitivity of MDA8 O_3_, we analyse MDA8 O_3_ under clear sky conditions for NO_*x*_ and VOC limited regimes separately. We do so to quantify the effect concerning changes in DA NO_*x*_ mixing ratio and *T*_mean_. Our results show that the temperature sensitivity of ozone formation increases wtih rising *T*_mean_. This pattern is found in spring and in summer. Still, the absolute temperature sensitivity is, on average, a factor of 2.5 larger in the summertime than in spring at all site categories (4.4–5.6 μg per m^3^ per °C *T*_mean_ in JJA). Besides enhanced chemical kinetics at elevated temperatures, increased BVOC emissions, reduced dry deposition rates, and faster PAN decomposition (NO_2_ source, specifically relevant in the NO_*x*_ limited regime) result in increased ozone concentrations. The enhancement of MDA8 O_3_ due to higher ambient temperatures is in the same order as the ozone reduction achieved due to cuts in anthropogenic precursor emissions over the last 30 years.

For the city of Vienna, we employ MAX-DOAS HCHO measurements (06/2017–12/2019) to evaluate whether BVOCs are the dominant source in the metropolitan area in spring and summer. Throughout the growing season (April–October), we find a distinct positive relationship between HCHO and temperature, indicative of the hydrocarbons' biogenic source. Additionally, we derive ozone isopleths by combining the MAX-DOAS HCHO measurements and *in situ* O_3_ and NO_*x*_ observations (Vienna, Stephansplatz). The isopleths confirm that peak MDA8 O_3_ is VOC limited.

In conclusion, our analysis shows that during the last three decades, rising air temperatures have been counteracting the effects of ozone precursor reductions and altering the NO_*x*_ to VOC ratios by modifying the contribution of BVOCs to the total VOC burden. In particular, the latter poses a challenge for air quality managers, given the temperature dependence of both BVOC emission and ozone production, in the light of progressive climate change. The assessment of potential changes in the future Austrian ozone burden – and the relative contribution of changes in temperature, and anthropogenic and biogenic precursor emissions to it – is left for future modelling work, following the representative concentration pathways.^53^

## Conflicts of interest

There are no conflicts to declare.

## Supplementary Material

EA-002-D2EA00004K-s001
