# Supplementary material for: An analysis of 30 years of surface ozone concentrations in Austria: temporal evolution, changes in precursor emissions and chemical regimes, temperature dependence, and lessons for the future
Source: Environ Sci Atmos. 2022 Apr 19;2(4):601–15. doi: 10.1039/d2ea00004k (PMC9281626; doi:10.1039/d2ea00004k)
Supplement: EA-002-D2EA00004K-s001 [file EA-002-D2EA00004K-s001.pdf]

Electronic Supplementary Material (ESI) for Environmental Science: Atmospheres.
This journal is © The Royal Society of Chemistry 2022

Supplementary information

Table S1: List of O_3 background sites of the Austrian monitoring network used in this study. Station printed in bold provide NO_x observations for the whole study period along with ozone measurements. Sites marked with * indicate the sites selected for representative analysis.

	Station name	category	Airbase code		Station name	category	Airbase code
**1**	**Amstetten**	**urban**	**AT30101**	29	Arnfels	rural	AT60190
2	Graz, Schlossberg	urban	AT60018	**30**	**Dunkelsteinerwald**	**Rural**	**AT31701**
**3**	**Leoben**	**urban**	**AT60143**	**31**	**Forsthof am Schöpfl**	**rural**	**AT30202**
**4**	**Linz, Neue Welt**	**urban**	**AT4S416**	**32**	**Gänserndorf**	**rural**	**AT30401**
**5**	**St. Pölten**	**urban**	**AT32301**	33	Grundlsee	rural	AT60157
**6**	**Vienna, Stephansplatz***	**urban**	**AT9STEF**	**34**	Haunsberg	rural	AT53055
**7**	**Bad Ischl**	**suburban**	**AT4S125**	**35**	**Heidenreichstein**	**rural**	**AT30502**
**8**	**Bad Vöslau - Gainfarn**	**suburban**	**AT30201**	36	Hochgössnitz	rural	AT60137
**9**	**Eisenstadt**	**suburban**	**AT10001**	**37**	Höfen Lärchbichl	rural	AT72705
**10**	**Graz Nord***	**suburban**	**AT60138**	**38**	**Illmitz***	**rural**	**AT0ILL1**
**11**	**Hainburg**	**suburban**	**AT30301**	39	Irnfritz	rural	AT30801
**12**	**Innsbruck Sadrach**	**suburban**	**AT72113**	**40**	**Klöch***	**rural**	**AT60185 (O_3)**
**13**	**Judenburg**	**suburban**	**AT60018**		**Klöch***	**rural**	**AT0KLH1(NO_x)**
14	Klagenfurt Kreuzbergl	suburban	AT2KA41	41	Kollmitzberg	rural	AT30103
**15**	**Klosterneuburg**	**suburban**	**AT30601**	**42**	**Kramsach Angerberg**	**rural**	**AT72538**
16	Lustenau Wiesenrain	suburban	AT80706	**43**	**Masenberg***	**rural**	**AT60156**
**17**	**Mödling**	**suburban**	**AT31401**	44	Mistelbach	rural	AT331301
**18**	**Spittal a.d.Drau**	**suburban**	**AT2SP18**	**45**	**Obervellach**	**rural**	**AT2SP10**
**19**	**St. Johann im Pongau**	**suburban**	**AT54057**	46	**Pillersdorf**	**rural**	**AT0PIL1**
**20**	**Steyr**	**suburban**	**AT4S409**	**47**	**St. Georgen /Lavanttal**	**rural**	**AT2WO35**
**21**	**Traun**	**suburban**	**AT4S404**	48	St. Koloman Kleinhorn	rural	AT52055
22	**Vienna, Hermannskogel***	**suburban**	**AT9JAEG**	**49**	**Stixneusiedl**	**rural**	**AT30302**
**23**	**Vienna, Hohe Warte**	**suburban**	**AT900ZA**	**50**	**Streithofen**	**rural**	**AT31904**
24	Vienna, Laaer Berg	suburban	AT90LAA	51	**Sulzberg - Gmeind**	**rural**	**AT80503**
**25**	**Vienna, Lobau**	**suburban**	**AT90LOB**	**52**	**Vorhegg**	**rural**	**AT0VOR1**
**26**	**Wiener Neustadt**	**suburban**	**AT332401**	**53**	Wiesmath	rural	AT32101
**27**	**Wolfsberg**	**suburban**	**AT2WO15**	**54**	**Wolkersdorf**	**rural**	**AT30403**
28	Annaberg	rural	AT31102				

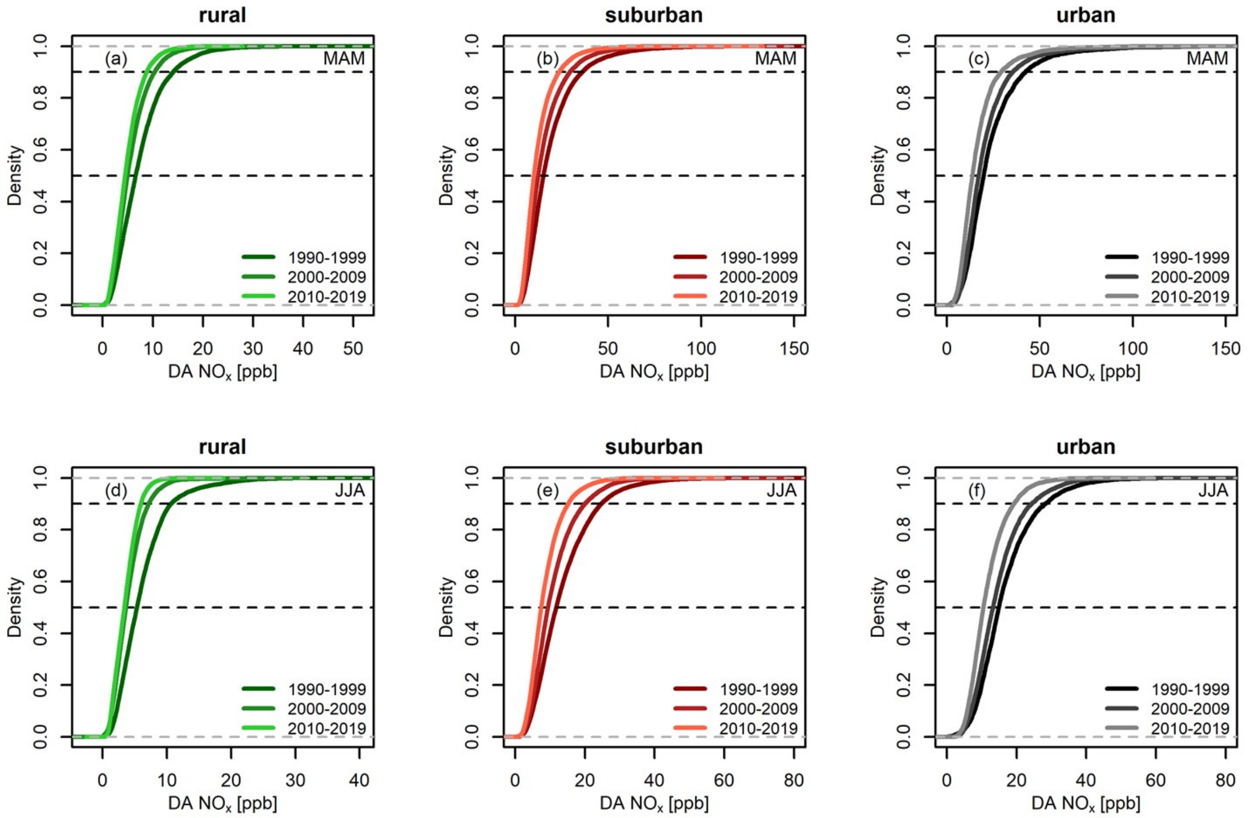

Figure S1: Empirical cumulative probability distribution functions (ECDFs) of daily average (DA) NO_x concentrations for (a) rural, (b) suburban, and (c) urban sites during spring (MAM). (d)-(f) as (a) – (c) but for summer (JJA). Dashed lines in all panels indicate the 50th and 90th percentiles.

Table S2: Ratios of selected NO_x percentiles (50% (q50) and 90%(q90)) for spring and summer seasons for different site types for 10 year study periods relative to 1990-1999.

Site category / quantile	MAM 2000-2009	MAM 2010-2019	JJA 2000-2009	JJA 2010-2019
Rural q50	0.76	0.68	0.68	0.63
Suburban q50	0.82	0.66	0.80	0.64
Urban q50	0.87	0.70	0.89	0.70
Rural q90	0.74	0.63	0.67	0.56
Suburban q90	0.82	0.65	0.81	0.62
Urban q90	0.84	0.69	0.86	0.68

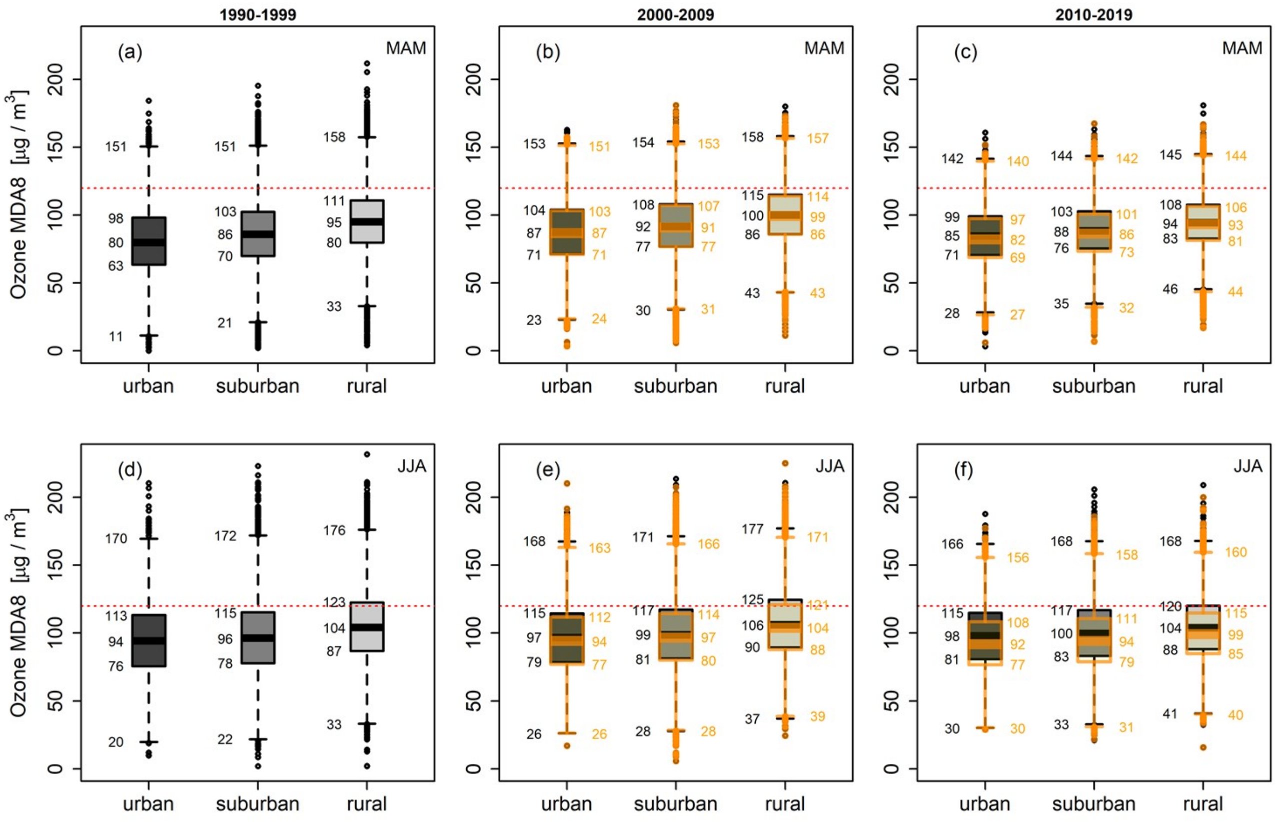

Figure S2: As figure 3 but for MDA8 O_3 concentrations.

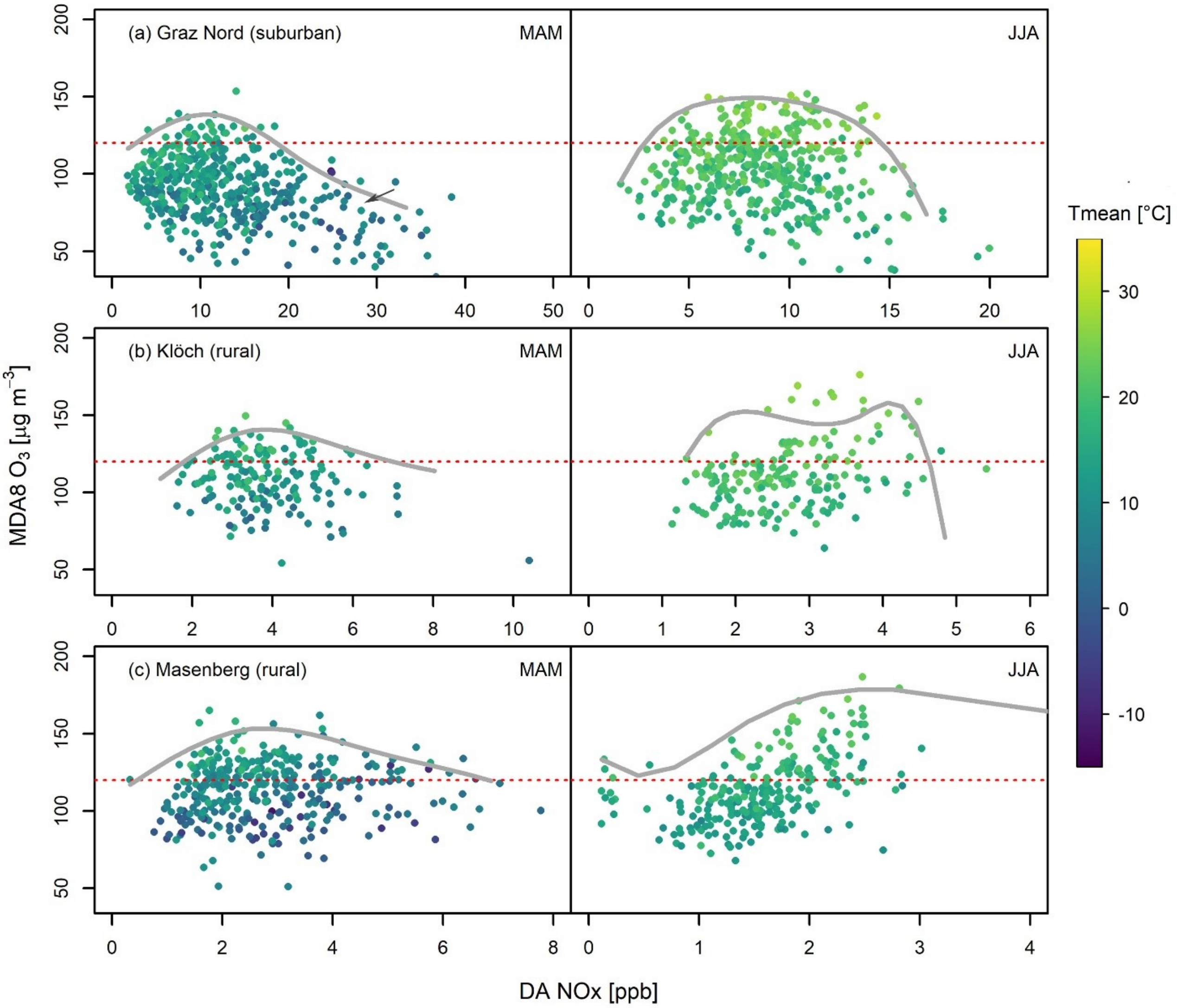

Figure S3: As figure 5 but for sites Graz (12% of observations NO_x limited in JJA), Klöch and Masenberg.

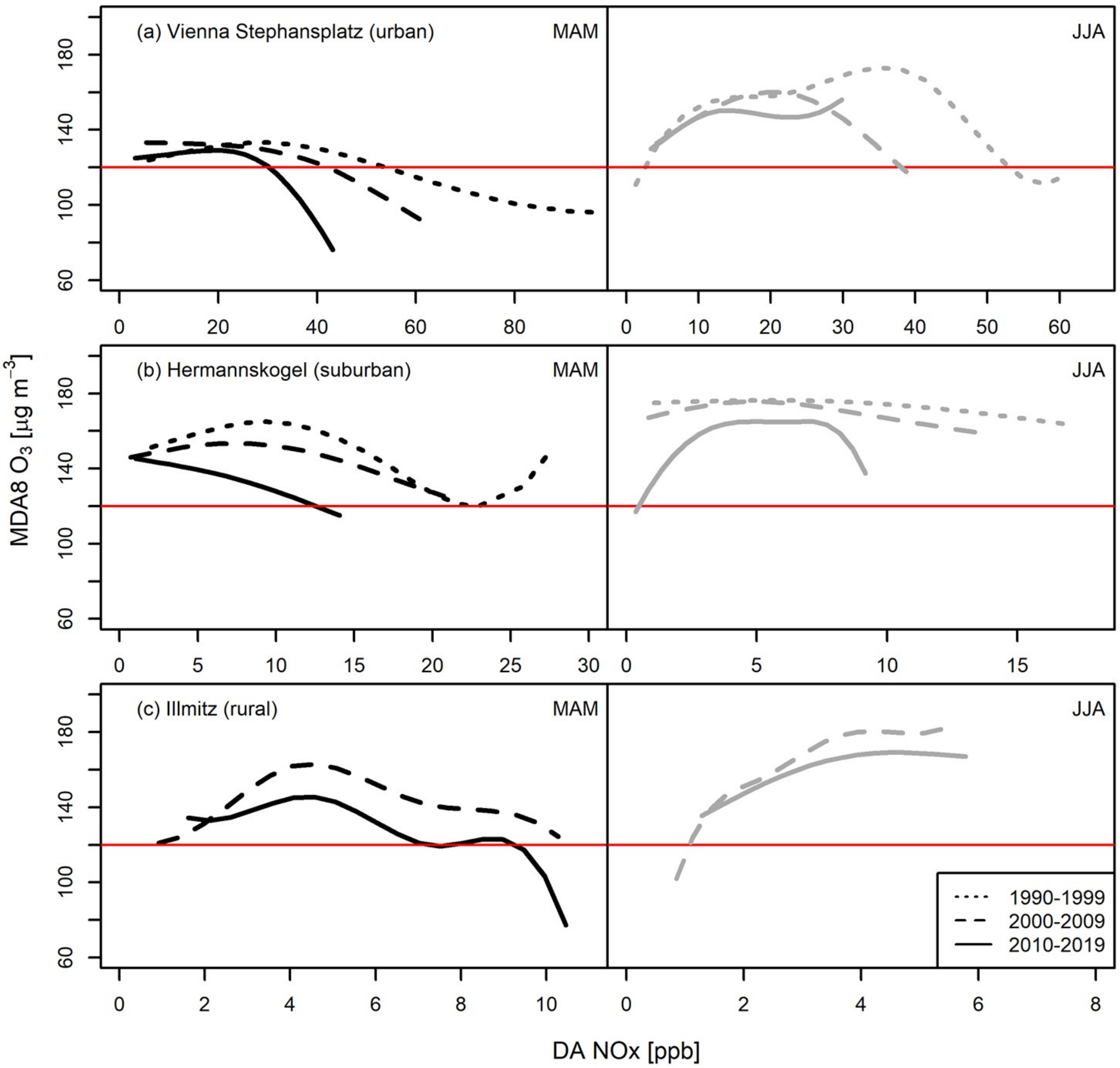

Figure S4: Evolution of ambient chemical regimes over time for (a) the urban station Vienna Stephansplatz, (b) the suburban site Hermannskogel, and (c) the rural site Illmitz; in analogy to Figure 5. The splines are calculated as polynomial fits of 4th order to the envelopes of the scatterplots of MDA8 O_3 and DA NO_x for the respective decadal time slices. Note, for the rural site Illmitz the period 1990-1999 is not evaluated as NO_x measurements started only in 1995.

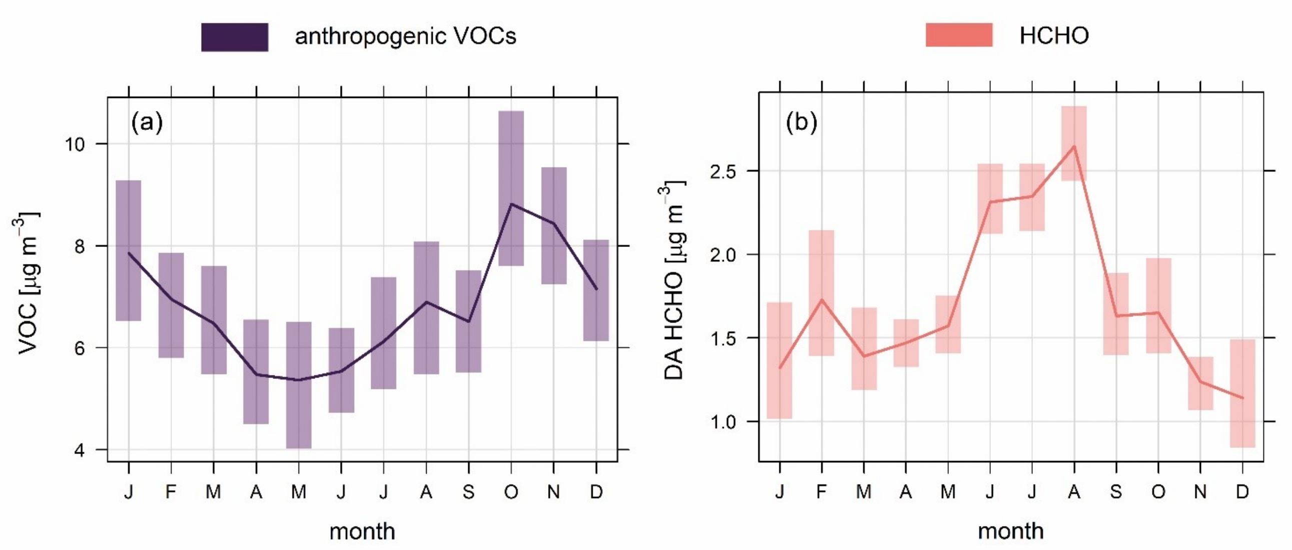

Figure S5: Annual cycle of (a) the sum of selected anthropogenic VOCs (iso-pentane, 1-pentene, n-pentane, 2-pentene, iso-hexane, n-hexane, benzene, iso-octane, n-heptane, toluene, n-octane, ethylbenzene, m-xylene, p-xylene, o-xylene, 1,3,5-Trimethylbenzol, 1,2,4-trimethylbenzene and 1,2,3- trimethylbenzene) and (b) HCHO in Vienna. Data from (a) are collected during 2017-2019 close to the centre of Vienna at the monitoring site AKH. The collection cycle is every 6th day, and samples are analysed by gas chromatography. Data in (b) are HCHO data obtained during 2017-2019 by the MAX-DOAS instrument at BOKU Vienna.

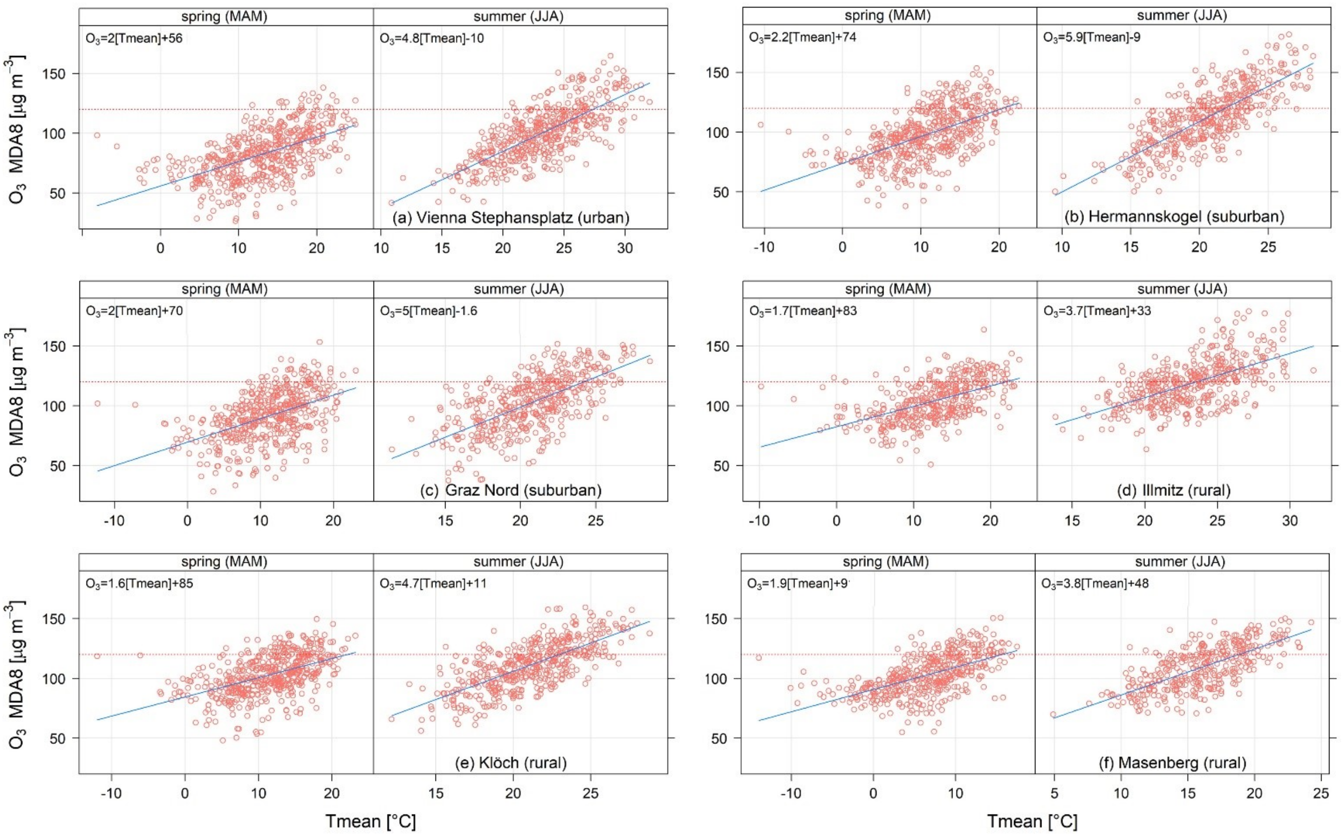

Figure S6: Scatterplots of MDA8 O_3 versus Tmean for (a) the urban station Vienna Center, (b)-(c) suburban sites Hermannskogel, and Graz, and (d)-(f) rural sites Illmitz, Klöch, und Masenberg for 20010 – 2019. The red dotted line in all panels indicates the MDA8 target value for the protection of human health of 120 µg/m^3, blue lines provide linear regression fits.

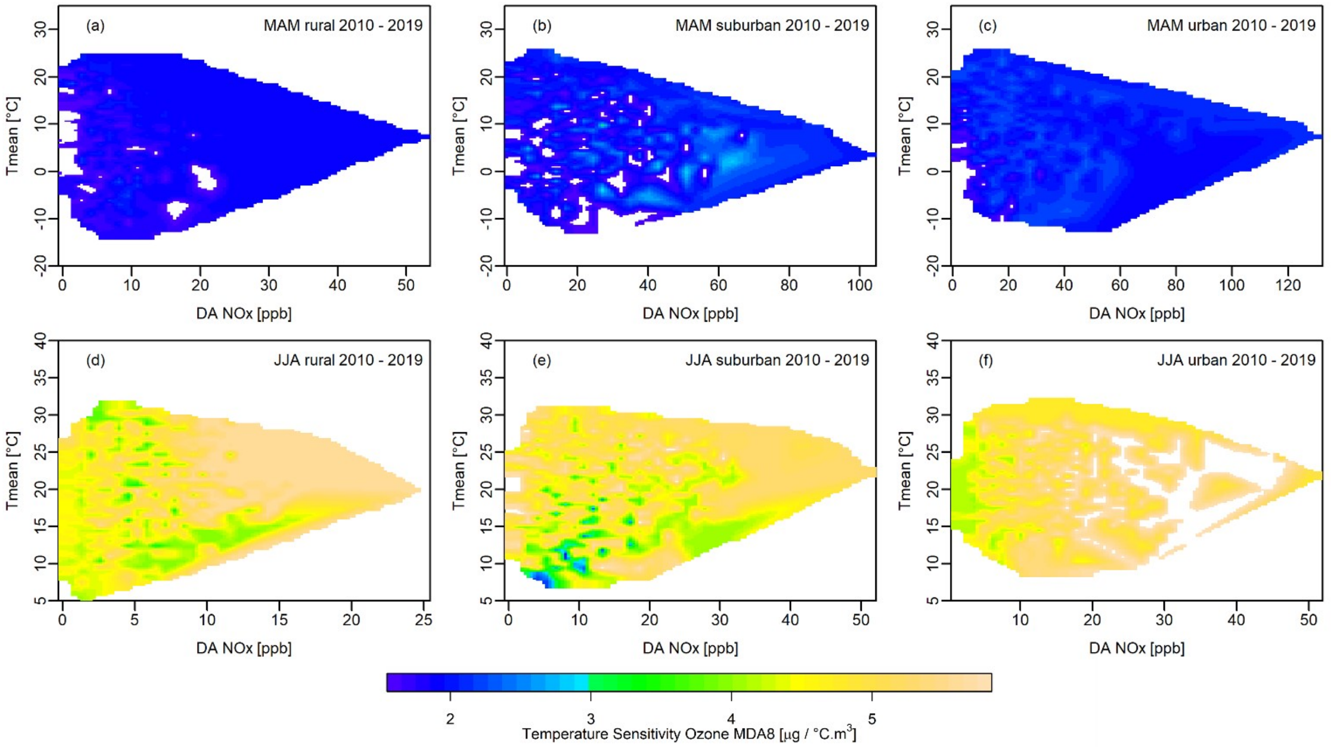

Figure S7: MDA8 ozone temperature sensitivities with respect to Tmean for rural, suburban, and urban monitors for various DA NO_x concentrations and mean temperatures. The ozone temperature sensitivities under clear sky conditions are shown for spring (MAM) in the top panel (a)-(c) and for summer (JJA) in the bottom panel (d)-(f). These data are used to compile the contour plots given in figure 7.

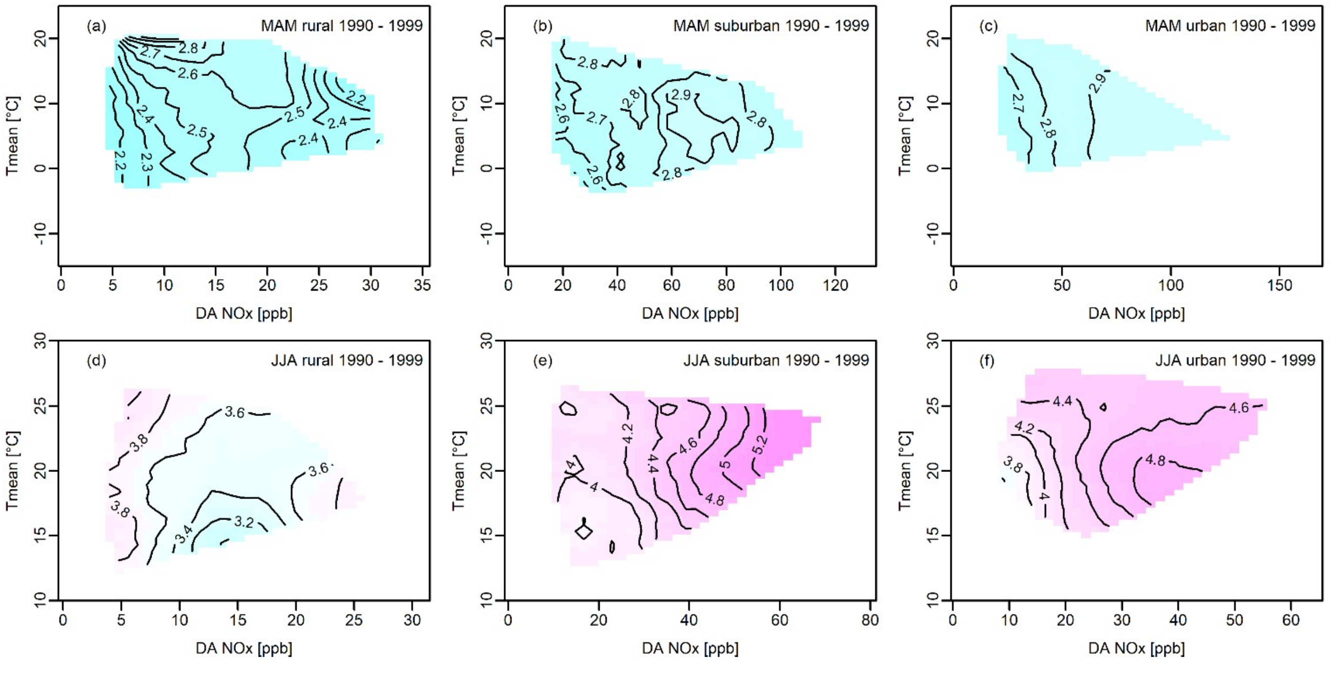

Figure S8: MDA8 O_3 temperature sensitivities with respect to Tmean for rural, suburban, and urban monitors for various DA NO_x concentrations and mean temperatures. The ozone temperature sensitivities for 1990-1999 under clear sky conditions are shown for spring (MAM) in the top panel (a)-(c) and for summer (JJA) in the bottom panel (d)-(f). The contours of the temperature sensitivity of ozone are given in µg/°C.m^3 with respect to changes of Tmean.

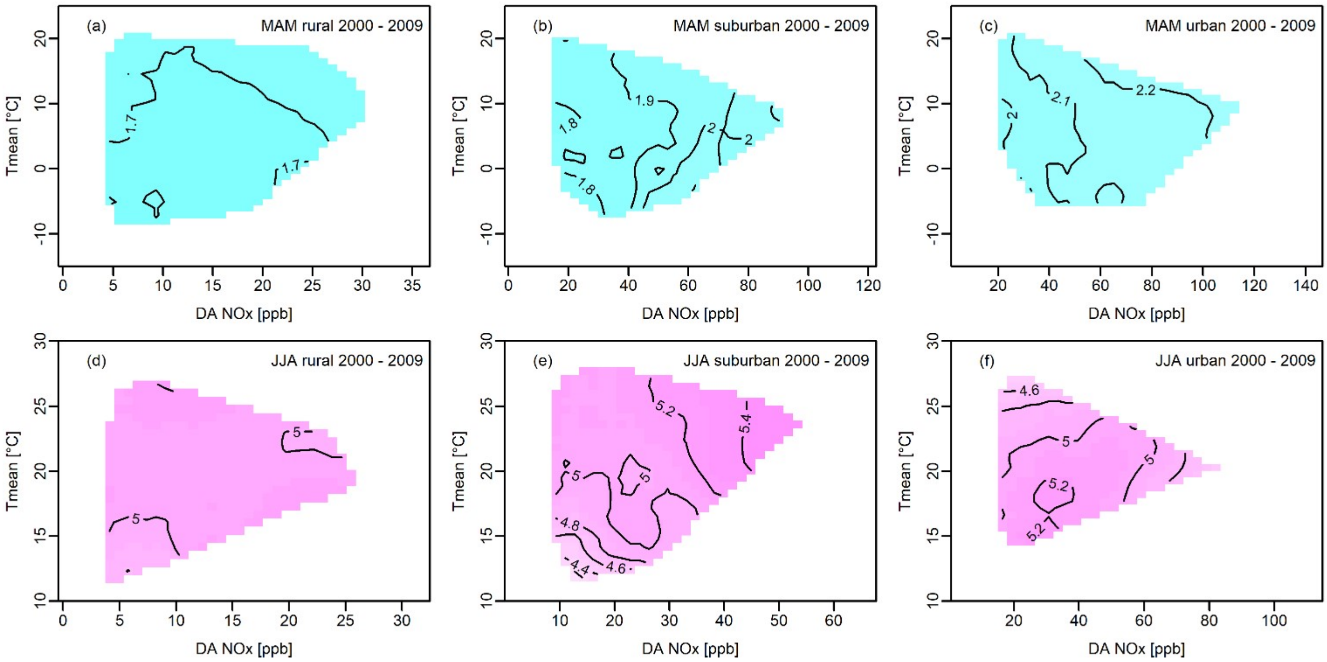

Figure S9: MDA8 O_3 temperature sensitivities with respect to Tmean for rural, suburban, and urban monitors for various DA NO_x concentrations and mean temperatures. The ozone temperature sensitivities for 1990-1999 under clear sky conditions are shown for spring (MAM) in the top panel (a)-(c) and for summer (JJA) in the bottom panel (d)-(f). The contours of the temperature sensitivity of ozone are given in µg/°C.m^3 with respect to changes of Tmean.
